# Burden of prehypertension among adults in Kenya: a retrospective analysis of findings from the Healthy Heart Africa (HHA) Programme

**DOI:** 10.1186/s12889-020-8363-z

**Published:** 2020-03-03

**Authors:** Jared O. Mecha, Elizabeth N. Kubo, Collins O. Odhiambo, Freda G. Kinoti, Kennedy Njau, Gerald Yonga, Elijah N. Ogola

**Affiliations:** 1 Department of Clinical Medicine and Therapeutics, University of Nairobi, Nairobi, Kenya; 2 Healthy Heart Africa, Nairobi, Kenya

**Keywords:** Prehypertension, Prevalence, Cardiovascular disease, Kenya, Sub-Saharan Africa

## Abstract

**Background:**

Hypertension is the leading risk factor for mortality globally. African countries, including Kenya, have a high and rising prevalence of hypertension. Prehypertension is associated with an increased risk of progression to overt hypertension and a higher risk of cardiovascular disease and mortality. Despite this, little is documented on the prevalence and distribution of prehypertension in sub-Saharan Africa. This study sought to estimate the overall burden of prehypertension in Kenyan adults enrolled in a large hypertension control programme, Healthy Heart Africa. The distribution and determinants of prehypertension in the sample were explored as secondary objectives.

**Methods:**

This was a post hoc analysis of cross-sectional data obtained from population-level blood pressure (BP) screening of adults aged ≥18 years in the community and ambulatory care facilities in 17/47 sub-national administrative units in Kenya. All participants with a complete record for systolic and diastolic BP were included. Descriptive analyses were performed for sociodemographic characteristics. Pearson’s chi-square test was used to assess differences in categorical variables. Multivariate logistic regression analysis was performed to identify factors independently associated with prehypertension.

**Results:**

Of 5,985,185 participant records that were included in the analysis, 34% were men (mean age: 45 [SD 2.9] years). The majority (63%) lived in rural Kenya. The prevalence of prehypertension was 54.5% and that of hypertension was 20.8%. Characteristics that were independently associated with prehypertension (adjusted odds ratio [95% CI]) included male sex (1.23 [±0.0023], *p* <  0.001 for all age groups > 25 years) and rural residence (1.60 [±0.023], *p* <  0.001).

**Conclusions:**

Approximately one in every two Kenyan adults has prehypertension. This calls for urgent development and roll-out of a national BP screening and control programme. It also provides a strong basis for the formulation of multisectoral national policies that will ensure implementation of evidence-based, low-cost public health interventions geared towards primary prevention of hypertension, especially in population groups that are traditionally considered at low risk, such as young adults and rural residents.

## Background

Raised blood pressure (BP) is the leading risk factor for global morbidity and mortality [1]. Africa has the highest age-adjusted prevalence of hypertension globally [2]. A meta-analysis of recent studies in Africa showed a prevalence of 30%, with a progressive rise with increasing age [3]. In Kenya, a recent national survey found a prevalence of 24.5% [4], consistent with the results of the May Measurement Month global screening exercise (MMM17) in which 24.6% of those screened were hypertensive [5]. Prehypertension as a BP category was introduced in 2003 by the Seventh Joint National Committee on Prevention, Detection, Evaluation and Treatment of High Blood Pressure (JNC-7) [6] to highlight the excess risk associated with BP in this range [7]. The excess risk associated with elevated BP begins at a systolic BP of 115 mmHg and a diastolic BP of 75 mmHg. Prehypertension is, therefore, not only associated with a higher risk of transition to overt hypertension but is also independently associated with a higher cardiovascular risk. Given the link between the continuum of prehypertension, hypertension, and ultimately cardiovascular disease morbidity and mortality, early identification of prehypertension may be a critical first step in increasing awareness and designing and implementing early preventive interventions.

Despite the public health threat posed, there is a paucity of data on the prevalence and distribution of prehypertension in sub-Saharan Africa. A skewed focus on infectious diseases, maternal and child health and acute care, compounded by weak health systems, has led to the general neglect of cardiovascular disease risk factor identification and mitigation [8]. Consequently, people with prehypertension frequently go undetected and are not followed up to prevent progression. The identification of prehypertension presents a relatively low-cost opportunity to initiate cardiovascular risk reduction for a large proportion of the population. Although two previous Kenyan studies have documented high prevalence of prehypertension (53 and 59%) [9, 10], there is a lack of general awareness on the need for screening and follow-up for prehypertension.

The primary objective of this study was to estimate the burden of prehypertension in a population of Kenyan adults who were enrolled in a large hypertension screening, treatment and control programme, Healthy Heart Africa (HHA). Secondary objectives were to explore the distribution and determinants of prehypertension in the study population.

## Methods

### Study design

This was a post hoc analysis of cross-sectional survey data obtained from a population-level BP screening campaign that was part of the HHA programme. HHA was conducted through a public-private partnership initiative between the Kenya Ministry of Health and AstraZeneca PLC, from February 2015 to October 2018.

### Study setting

Details of the HHA programme have been published previously [11]. In summary, the programme was implemented in 17 out of a possible 47 Kenyan counties between March 2015 and March 2016. The main goal of the HHA programme was to reduce barriers to hypertension screening, identification, referral and treatment, through several approaches, including: (1) education and raising awareness of hypertension among healthcare workers (HCWs) and the general public; (2) opt-out facility and community-based screening for hypertension by trained lay providers, especially in non-traditional screening settings such as religious and commercial centres; (3) training of HCWs on the Kenya Ministry of Health-approved hypertension screening and treatment protocol in order to enhance hypertension diagnosis and treatment; and (4) provision of a consistent supply of quality-assured medicines for hypertension treatment.

Standardised registers were used to collect participant information. These data were checked for accuracy, de-identified and uploaded onto a central database monthly.

### Participants

The study population was drawn from health facilities and their respective catchment communities. Health facility–based participants comprised adult patients aged 18 years and above who were seeking routine ambulatory health services. Community-based participants were adults aged 18 years and above who volunteered for BP screening at strategically located booths at non-clinical community settings. Trained laypersons conducted the BP screening using standardised protocols [12]. At a minimum, the trained laypersons were high school graduates. Briefly, BP was measured using CE marked Omron M3 digital devises (Omron Healthcare, Kyoto, Japan) with the participant seated in an upright, relaxed position. An appropriate cuff size was selected for each participant, and the cuff was wrapped around the left arm, supported at the level of the heart. Two readings were taken 2 min apart, and the average of the two was recorded. To ensure reliability and validity of the measurements, the lay field assistants received standardised training on BP measurement and the use of standard operating procedures. Trained nurses also carried out regular supportive supervision and provided mentorship and support.

All participants who had a recorded systolic and diastolic BP were included in the analysis. Out of 5,985,185 participants who were screened, 5790 and 6861 had missing systolic and diastolic BP readings, respectively, and were therefore excluded from the analysis.

### Data handling

The de-identified analysis database was obtained from the central database in a CSV command delimiter, UTF-16 LE file encoding format. Data were then imported to RStudio using the *read.delim* function. Data cleaning, decoding, recoding and analysis were performed using RStudio.

### Variables

The main outcome variable of interest was prehypertension. This was defined as a systolic BP of 120–139 mmHg and/or diastolic BP of 80–89 mmHg [6]. Other outcomes of interest were normotension and hypertension. Normotension was defined as a systolic BP ≤120 mmHg and a diastolic BP ≤80 mmHg [6]. Hypertension was defined as a systolic BP ≥140 mmHg and/or a diastolic BP ≥90 mmHg [6].

Predictor variables included age (categorized into six age groups: 18–25, 26–35, 36–45, 46–55, 56–65 and 65+ years), sex and place of residence (urban/rural).

A negligible number of participants had missing data for the various variables of interest and were therefore omitted from the final analysis.

### Statistical analysis

Descriptive analyses were performed for sociodemographic characteristics of the study population. Categorical variables were presented using proportions. Continuous variables were presented using means with corresponding standard deviations (SDs). Pearson’s chi-square test was used to assess differences in categorical variables. Multivariate logistic regression analysis was performed to identify factors independently associated with prehypertension. Crude and adjusted odds ratios (aORs) are presented. All statistical tests were two-sided, and *p* values < 0.05 were considered statistically significant. All analyses were performed using RStudio (2015), RStudio: Integrated Development for R (RStudio, Inc., Boston, MA).

## Results

Out of a total of 5,985,185 participant records that were analysed, 2,057,674 (34%) were men. The overall mean age was 45 years (SD 2.9). Men had a mean age of 47 years (SD 1.13) and were older than women who had a mean age of 43 years (SD 1.7). Approximately 63% of the participants were rural dwellers.

### Burden of abnormal blood pressure

The distribution of prehypertension and hypertension by participant characteristics is presented in Table 1.

**Table 1:** Burden of prehypertension and hypertension by participant characteristics (*n* = 5,985,185)

Characteristics	Normal	Prehypertension	Hypertension
	*N* = 1,478,920	*N* = 3,262,387	*N* = 1,243,878
Prevalence, % (CI)	24.7 (±0.38)	54.5% (±1.39)	20.8 (±0.2)
Age (years)			
mean (SD)	39 (±0.02)	46(±0.02)	51(±0.02)
Age group, %, (CI)			
18–25,*n* = 767,730	45%(±0.1)	47%(±0.3)	8%(±0.6)
26–35, n = 1,488,388	35%(±0.1)	53%(±0.2)	12%(±0.1)
36–45, n = 1,305,740	25%(±0.4)	57%(±0.3)	18%(±0.3)
46–55, *n* = 964,863	15%(±0.5)	58%(±0.6)	27%(±0.4)
56–65, *n* = 645,912	9%(±0.3)	57%(±0.4)	34%(±0.5)
65+, *n* = 534,324	9%(±0.2)	63%(±0.3)	28%(±0.4)
Sex, % (CI)			
male, *n* = 2,057,694	19%(±0.5)	59%(±0.2)	22%(±0.3)
female, n = 3,903,062	28%(±0.1)	52%(±0.1)	20%(±0.1)
Residence, %, (CI)			
Urban, *n* = 468,820	22%(±0.3)	55%(±0.2)	24%(±0.4)
Rural, *n* = 843,944	14%(±0.3)	57%(±0.2)	29%(±0.2)

The overall burden of prehypertension was 54.5% and that of hypertension was 20.8%. The prevalence of prehypertension was higher among men (59%) compared to women (52%) (*p* <  0.001). Similarly, the burden of hypertension was higher among men (22.0%) compared to women (20%) (*p* <  0.001). The highest proportion of prehypertension (63%) was recorded among those aged 65 years and older. More rural residents had prehypertension compared to urban dwellers (57% vs 55%, respectively) (*p* <  0.001). Likewise, more rural dwellers had hypertension compared to their urban counterparts (29% vs 24%, respectively) (*p* <  0.001).

### Burden of prehypertension by age group

**Fig. 1:**
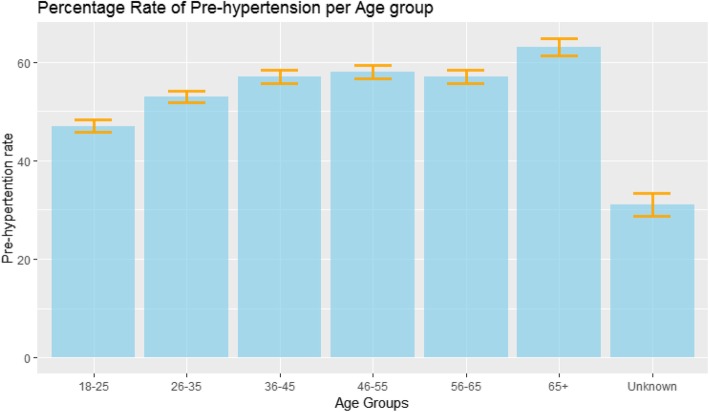
Burden of prehypertension by age group

Figure 1 shows the proportion of prehypertension by age group. Overall, the burden increased with increasing age.

### Independent predictors of prehypertension

Independent predictors of prehypertension are presented in Table 2. Characteristics that were independently associated with prehypertension (aOR [95% confidence interval]) included male sex (1.23 [±0.0023], *p* <  0.001 for all age groups > 25 years) and rural residence (1.60 [±0.023], *p* <  0.001).

**Table 2:** Characteristics associated with prehypertension

Variable	OR (95% CI)	*p*-value	aOR (95% CI)	*p*-value
Sex				
Female	ref	ref	ref	ref
Male	1.245(±0.0017)	< 0.001	1.229 (±0.0023)	< 0.001
Age group (yrs.)				
factor(Age group) 18–25	ref	ref	ref	ref
factor(Age group) 26–35	1.516(±0.015)	< 0.001	1.411(±0.0019)	< 0.001
factor(Age group) 36–45	1.661(±0.016)	< 0.001	1.523(±0.0015)	< 0.001
factor(Age group) 46–55	1.624(±0.016)	< 0.001	1.611(±0.0017)	< 0.001
factor(Age group) 56–65	1.575(±0.015)	< 0.001	1.521(±0.0017)	< 0.001
factor(Age group) 65+	1.483(±0.015)	< 0.001	1.480(±0.0017)	< 0.001
Residence				
urban	ref	ref	ref	ref
rural	1.645(±0.018)	< 0.001	1.599 (±0.023)	< 0.001

## Discussion

HHA was the largest hypertension screening program to be conducted in Kenya, and was planned to raise awareness on hypertension along with providing education, training and treatment. This study demonstrated that approximately one in every two Kenyan adults has prehypertension. The condition is more common among men than women, and the burden increases with increasing age. The rise begins very early in the third decade of life, a population often overlooked by hypertension control programmes. This is a worrying finding in view of the excess cardiovascular risk associated with prehypertension and underscores the need for early screening programmes and population-wide primary prevention measures. Previous research has documented a 27% increase in all-cause and a 66% increase in cardiovascular disease mortality associated with prehypertension [13]. The high burden of prehypertension documented here is also of concern in view of the associated risk of transition to hypertension. Identification of prehypertension thus presents a significant opportunity to improve the cardiovascular risk of a significant proportion of the population.

Although the burden of prehypertension documented here is within the 53–59.3% range previously reported in Kenya [9, 10], it is higher than some of the reports in the literature which showed a prevalence of 31–48.9% in non-African contexts. Our findings are consistent with growing evidence that, globally, Africa has the highest rates of age-adjusted prevalence of hypertension [14–19].

Similar to prior studies, men were more likely to have prehypertension than women [15, 17–21]. This may partly be related to the protection rendered to women by hormonal factors as well as pregnancy and child birth–related factors [22].

Our analyses found a slightly higher proportion of prehypertension among rural residents. This could be attributed to lower rates of screening and diagnosis among rural compared with urban residents. The Kenya STEPwise Survey documented that 60.7% of rural residents had never measured their BP, compared with 48.1% of urban residents [23].

Overall, these findings call for the urgent launch of a national BP control programme to formulate and implement multisectoral evidence-informed public health interventions geared towards the primary prevention of hypertension. Such interventions that are centred on lifestyle modifications would include physical activity, healthy diets rich in fruits and vegetables, low fat dairy products, reduced saturated fats and reduced dietary salt intake [24]. Currently, there is no evidence for pharmacological therapy for prehypertension except in very high risk patients.

The main strength of this study is the regional distribution of study participants, with representation from more than a third of all the counties in Kenya. This allows for in-country generalisability of findings and provides a strong evidence base to inform discussions on policies and guidelines for preventing cardiovascular disease risks.

Our study had a number of limitations, mostly inherent to the study design. First, due to the retrospective nature of the study, data on crucial known risk factors for prehypertension, including smoking, alcohol intake, body mass index, physical activity and dietary factors, were not collected. These were therefore not included in the regression model, hence affecting control for confounding. Second, the HHA data collection was cross-sectional. This implies that BP classification was based on readings that were recorded during a single sitting per participant. This could have resulted in an overestimate of prehypertension, as some of the elevated BP recordings documented within facility settings could have been the result of transient elevations [25]. Finally, although we report on about 6 million participants, the programme participants were drawn from a convenient sample in the context of a population BP screening programme. However, the distribution of BP was similar to previous national representative survey [23].

## Conclusions

Approximately one in two Kenyan adults has prehypertension, and it starts at a very young age. This calls for urgent public health mitigation strategies in order to avert prehypertension-associated morbidity and mortality. Further studies are needed to better define the role of traditional and novel anthropometric, biochemical and environmental risk factors in the causation of prehypertension and to identify the determinants and rate of regression of prehypertension to normal BP or regression to overt hypertension.

## Data Availability

All data generated or analysed during the current study are available from the sponsoring agencies on request though the corresponding author.

## Abbreviations

aOR: Adjusted odds ratio
BP: Blood pressure
HCW: Healthcare worker
HHA: Healthy Heart Africa
SD: Standard deviation
